# THE RIGHT STRATEGY, THE RIGHT PERSON, THE RIGHT TIME: DEVELOPING AN ADVOCACY FIELD GUIDE FOR RURAL GERIATRICS

**DOI:** 10.1093/geroni/igae098.0980

**Published:** 2024-12-31

**Authors:** Emily Franzosa, Lynette Kelley, Eileen Dryden, Lauren Hall, Lauren Moo, William Hung

**Affiliations:** James J Peters VA Medical Center, Bronx, New York, United States; Eastern Colorado Health Care System Rocky Mountain Regional VA, Aurora, Colorado, United States; Veterans Health Administration, Bedford, Massachusetts, United States; James J Peters VA Medical Center, Bronx, New York, United States; Department of Veterans Affairs, Bedford, Massachusetts, United States; James J Peters VA Medical Center, James J Peters VA Medical Center, New York, United States

## Abstract

The need to expand rural geriatric services has received increasing attention within and outside the VHA, yet medical center leaders are often challenged to prioritize expanding these services among myriad competing needs. To better understand potential levers to move rural geriatrics up the priority ladder, we conducted semi-structured interviews with 11 former leaders in the Veterans Health Administration. We explored perspectives on how health system priorities can be aligned to improve the quality and reach of rural geriatric care and solicited best practices for advocating for rural geriatric care at the local, regional and national level. Leaders shared that there was no ‘best way’ to advocate, prompting the development of an advocacy field guide to advance rural geriatric services. The guide draws from our interview data, policy analysis, and best practices in communication and advocacy to help local leaders and champions make a case for expanding rural geriatric resources. The guide describes multiple avenues to purposefully employ “the right strategy, for the right person, at the right time”, including data from mapping local and regional gaps and needs for services, storytelling, relationship building, building a business case, aligning rural geriatrics with regional and national goals, and making geriatrics “personal”. Providing actionable, easy-to-use resources to local champions can support efforts to expand rural geriatric services locally and nationally.

